# Education of Mothers

**Published:** 1883-11

**Authors:** 


ARTICLE III.

### EDUCATION OF MOTHERS.

BY MRS. M. W. J.

The reception of the July number of your *Journal*, which you so kindly sent me, has afforded great satisfaction, as I find that, although its pages are crowded with so much that must be of far greater interest to the gentlemen of your profession than anything I could say, you nevertheless made room for what I rather jestingly wrote you concerning what ladies expect from their dentists. Having met with so favorable a reception, however, I again venture to address you on another branch of the same subject, for it strikes me now that I omitted one very important thing that ladies, or perhaps I should say *mothers* expect, but which they too seldom receive, namely advice and instruction from their dentist as to the care of their own or their children's teeth.

If a child has the toothache, it is taken to the dentist, and the tooth is at once extracted, if the consent of the little one can be gained; if the case be otherwise, perhaps some trifling application is made which temporarily allays the pain until a renewed attack of suffering, with sleepless nights for all in the house, renders imperative another visit to the dentist, when the tooth is extracted, *nolens volens*, and they go their way rejoicing in the probable exemption from suffering for awhile at least, or until the next tooth aches.

There is "a little black spot" on one, perhaps, but that does not amount to anything. The dentist saw it, of course, but, for fear perhaps of seeming to "ask for a job," said nothing. "It is not for me to propose to do their work; they must come to me for my services; I cannot offer them"—and in a few weeks the child is brought back to have another tooth extracted. "It did not seem to be much decayed, doctor, but she was *cracking nuts* and the tooth just crushed all to pieces, and she has had a jumping toothache ever since!" Naturally enough, as the nerve is exposed, a piece of the nut-shell still lying on it.

So another tooth is extracted. The upper front teeth are seen to be disfigured with dark stains; the lower ones are covered with tartar; the jaw-teeth are imbedded in the foul, decaying remains of many previous meals, but no advice being asked, none is offered. The mother probably supposes that as these are "only baby teeth" which must evenall be *shed*, no care of them is necessary, and the sooner they are all extracted and out of the way the sooner will the child be freed from a necessary evil.

It is not my place to say anything here of the possible evil results of the premature extraction of the child's teeth; of the crowded, irregular, aud consequently early-decaying permanent teeth; or of the possible indigestions and dyspepsia from want of proper mastication, if the jaw-teeth are sacrificed; but that much of this terrible suffering, this loss of food and sleep, this *martyrdom of our babes* can be, and ought to be prevented, is an undoubted fact.

No child should be allowed to have a decayed tooth (sufficiently so to ache), and no mother should be allowed to remain in ignorance of the means by which this result can in a majority of cases be secured.

This perhaps appears rather strong langurge for me to use; but when I feel strongly, I must speak strongly.

Naturally anxious for the best welfare of her child, physically as well as mentally and morally, well-meant advice, kindly proffered, couched in proper terms, coming from a competent source, will never be rejected by any sensible mother.

Of course, it will do to state in plain terms the unpalatable truth: "The foul condition of your child's mouth, and its consequent suffering and misery, are the results of your own gross ignorance and criminal neglect."

The dentist, if a true gentleman, which every dentist should be, can readily convey this truth home to the mother's heart in language kind and sympathetic, and so impress it that his advice will be gratefully received and his instructions carefully followed.

Let him so win her confidence that she will look upon him in the same light that she does her old family physician who presides at the birth of every child, and as she consults him in regard to her own health and that of her children, so will she learn to consult her dentist as to the formation, growth and care of their teeth.

If proper advice were given every prospective mother regarding the care of herself, especially in regard to furnishing abundance of proper nutrient elements, "bone and tooth food," from the very hour of conception, children would be born with the tooth-germs so well nourished during fœtal life that they would erupt at the proper time with little or no disturbance, and they would be of such fine structure that but little care beyond strict cleanliness and proper diet would be required to keep them sound and perfect.

To attain this most desirable end, however, mothers must be taught how much depends upon their own efforts, rightly guided by the wise instructions of those made competent to guide and instruct by a lifetime of research and study.

The mechanical skill to patch up defects, or the artistic perception necessary to adapt artificial substitutes are all very well in these places, but how much nobler work, how far grander a boon to humanity is the ability to prevent these evils!

Teach mothers that the teeth are not formed, as so many evidently suppose, during the few weeks or months preceding eruption, when the gums are swollen, and the child cross and peevish, but that they date their existance almost from the very beginning of fœtal life; that as early as the sixth or seventh week after conception the germs of the teeth are forming in the dental groove—soft and pulpy, it is true, until about the fourth month, when calcification begins, the whole tooth being thoroughly solidified and the enamel formed before it makes its appearance in the baby's mouth, except that the root continues to elongate.

As the teeth can only be formed from tooth material, and as this is required from the very earliest beginning of the germ formation, teach the mother that she alone can and must supply this material. If she does not furnish it, designedly or otherwise, in sufficient quantity over and above the amount requisite for her own use, it will be subtracted from her own osseous tissues, and she will suffer correspondingly, not alone in her teeth and bones, under very insufficient regime even the brain will become enfeebled from lack of phosphoric acid, the muscles pale and flabby, and the poor mother absolutely famish for lack of the necessary elements of nutrition, even while apparently enjoying the most luxurious diet.

Teach the mother what this tooth-making material is, and where she is to find the necessary implements. Teach her that she must not only have proper food, and sufficient food, but that her system must be kept in condition to digest and assimilate this food. Teach her the importance of physical exercise, of fresh air and sunlight, and of cleanliness, as indispensable adjunts to diet.

Teach her that these principles must be applied and these precepts acted upon, not only through the nine months of *gestation*, while she supplies all the elements of nutrition through her blood, but also during the whole period of *lactation*, when her milk is only the sole magazine of lime-salts for the further development of the teeth and bones, but the only source of nutriment for the whole body of the rapidly-growing child.

If after weaning she will habituate her child to plain, wholesome food—and by this time you will have taught her what this means—with scrupulous cleanliness and abundant exercise for the organs of mastication : provide it with comfortable, easy dress, and enforce strict obedience to the laws of health, what a splendid race of men and women would we see in the next generation !

In the words of Dr. Welcheus, " Good, substantial food, containing all the elements necessary to build up and nourish the various tissues of the body—clean, warm clothing to protect the surface, and regular out-door exercise, all with temperance and moderation, will not only raise the child well, but, in a large variety of cases, *raise a denture* well calculated to withstand the changes of life, and endure the wear and tear oi mastication. Mothers and children would thus attain a higher standard of physical development, for these benefits could not accrue solely to *the teeth.* "A knowledge and observance of nature's laws must result in an improvement of the whole being, body, mind and heart."

And not only this, you would also have the proud satisfaction of feeling that you had done your share toward raising the dental profession to a higher standard of physiological science.—*Southern Dental Journal.*

